# *In situ* single-atom array synthesis using dynamic holographic optical tweezers

**DOI:** 10.1038/ncomms13317

**Published:** 2016-10-31

**Authors:** Hyosub Kim, Woojun Lee, Han-gyeol Lee, Hanlae Jo, Yunheung Song, Jaewook Ahn

**Affiliations:** 1 Department of Physics, KAIST, Daejeon 305-701, Korea

## Abstract

Establishing a reliable method to form scalable neutral-atom platforms is an essential cornerstone for quantum computation, quantum simulation and quantum many-body physics. Here we demonstrate a real-time transport of single atoms using holographic microtraps controlled by a liquid-crystal spatial light modulator. For this, an analytical design approach to flicker-free microtrap movement is devised and cold rubidium atoms are simultaneously rearranged with 2*N* motional degrees of freedom, representing unprecedented space controllability. We also accomplish an *in situ* feedback control for single-atom rearrangements with the high success rate of 99% for up to 10 μm translation. We hope this proof-of-principle demonstration of high-fidelity atom-array preparations will be useful for deterministic loading of *N* single atoms, especially on arbitrary lattice locations, and also for real-time qubit shuttling in high-dimensional quantum computing architectures.

## Abstract (web summary)

It would be desirable to have a reliable and scalable method to manipulate neutral-atoms for the creation of controllable quantum systems. Here the authors demonstrate real-time transport of single rubidium atoms in holographic microtraps controlled by liquid-crystal spatial light modulators.

## Graphical abstract

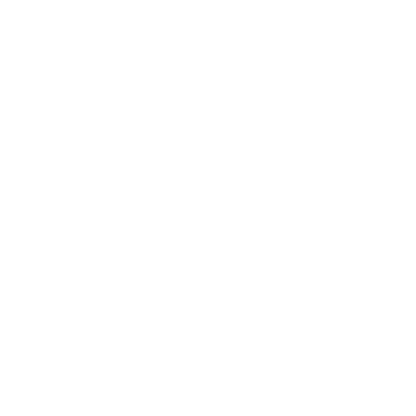

Laser cooling and trapping of atoms has enabled the construction and manipulation of quantum systems at the single-atom level^1^,^2^,^3^,^4^,^5^,^6^,^7^,^8^,^9^. To create scalable and highly controllable quantum systems, for example, a large-scale quantum information machine, further development of this bottom-up approach is necessary. The implementation of these systems has crucial prerequisites: scalability; site distinguishability; and reliable single-atom loading onto sites. The previously considered methods^10^,^11^,^12^,^13^ satisfy the two former conditions relatively well; however, the last condition, loading single atoms onto individual sites, relies mostly on probabilistic loading, implying that loading a predefined set of atoms at given positions will be hampered exponentially. Two approaches are readily thinkable to overcome this issue: increasing the single-atom loading efficiency^14^,^15^,^16^,^17^ and relocating abundant atoms to unfilled positions^18^,^19^. Realizing the atom relocation idea, in particular, is directly related to how many atoms can be transportable independently and simultaneously.

In this regard, using holographic optical tweezers is advantageous because arbitrary potentials can be designed for atoms^6^,^12^. The optical tweezers are the image determined by the wave propagation integral, for example, Fourier transformation, so the hologram for a complex potential can be designed using a numerical method often based on iterative Fourier transformation algorithms (IFTAs)^12^. When being used in conjunction with an active holographic device such as spatial light modulators (SLM), the algorithms can produce dynamic optical potentials, of which the many applications include dynamic *in situ* atom manipulation^20^,^21^,^22^,^23^, quantum logic gate^24^, pattern formations in an addressable optical lattice^19^ and real-time feedback transportation of atoms^7^. However, there has been no scheme implemented for the rearrangement of many atoms, with the full degree of control (2*N* for *N* atoms) in two-dimensional (2D) space.

In this paper, we demonstrate a dynamic holographic single-atom tweezer with unprecedented degrees of freedom of 2*N*. In a proof-of-principle experiment conducted with cold ^87^Rb atoms, simultaneous rearrangements of up to *N*=9 single atoms were successfully performed. This method may be further applicable to deterministic *N* single-atom loading, coherent transport^20^,^21^ and controlled collisions^22^,^23^.

## Results

### Experimental concept and flicker-free beam steering algorithm

**Figure 1:**
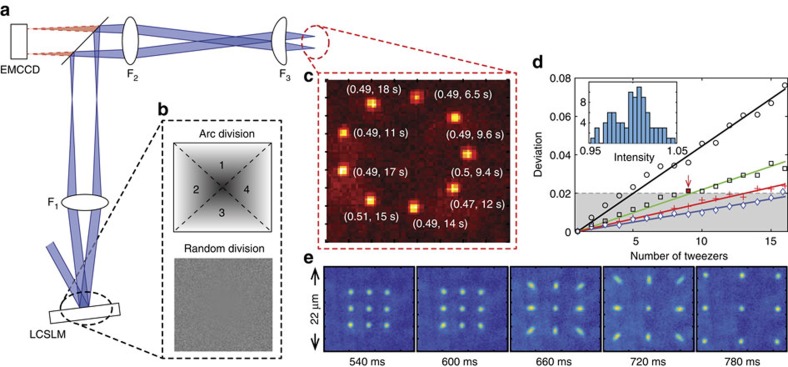
Setup for single-atom holographic transport. (**a**) The optical system for hologram transfer and trap imaging. (**b**) Schematic representation of 2D phase planes of the LC-SLM on the grey scale. One of them intuitively illustrates the working principle (arc division), and the other shows real hologram (random division), respectively. (**c**) An example of loaded single atoms in a tweezer array, represented with 22 × 22 μm^2^ size 500 cumulative images. The parenthesis denotes the loading probability and lifetime. (**d**) The intensity standard deviation as a function of *N*_t_ for the random division. The data points were calculated for various beam waists *W* at the SLM window (circles for *W*=1 mm, squares for 2 mm, crosses for 3 mm and diamonds for 4 mm) and the solid lines are the linear fits to the data. The inset presents the intensity histogram of *W*=2 mm for *N*_t_=9 of the red mark. (**e**) An *in situ* single-atom array expansion movie.

Figure 1 shows the concept of our experiment and the first demonstration of *N* single-atom transport with 2*N* motional degrees of freedom. The setup consists of an active holographic device (liquid-crystal SLM (LC-SLM)), an imaging system and a cold ^87^Rb atom chamber, as shown in Fig. 1a. When the holograms were transferred by a two-lens system to the entrance pupil of a high numerical aperture (NA=0.5) lens^12^, the optical tweezers had a beam radius of *w*_o_=1.14 μm and the trap frequencies were measured as *f*_*r*_=100 kHz and *f*_*z*_=17 kHz for radial and axial directions, respectively^25^. The temperature of the trapped atoms was measured as *T*=110(10) μK using the release and recapture method^26^, where the error was the 1*σ* band of each optical tweezer.

Schematic examples of our trap generation algorithm are represented in Fig. 1b. Our algorithm uses the simplest analytic form of beam steering, that is, the linear phase *ϕ*(*x*)=*k*_*x*_*x*. This phase modulation directly couples the modulation plane (Fourier domain) control parameter, *k*_*x*_, to the image plane position, *X*=*Fk*_*x*_/*k*, with one-to-one correspondence, where *F* is the lens focal length and *k* is the wave-vector of the trap light. Then, the given function becomes a flicker-free solution, because a linear combination of two phases, *αk*_1_*x*+(1−*α*)*k*_2_*x* with *α*∈[0, 1], is again a linear phase which smoothly sweeps the two focal points, *X*_1_=*Fk*_1_/*k* and *X*_2_=*Fk*_2_/*k* (see Methods for more details). To obtain more than a single optical tweezer, the modulation plane is divided into several sub-planes as in Fig. 1b^27^. When each sub-plane is assigned to each linear phase and the division is randomized in the single-pixel resolution of the device, this method effectively preserves the diffraction limit of the individual optical tweezers focused onto the image plane. Note that the required trap laser power in this manner scales with 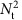, where *N*_t_ is the number of optical tweezers; hence, it is inefficient compared with the IFTA, which scales with *N*_t_ because the IFTA coherently sums over the entire modulation plane for every tweezer. Nevertheless, the proposed algorithm concedes power efficiency for independent controllability.

### Simultaneous transport of *N* single atoms

**Figure 2:**
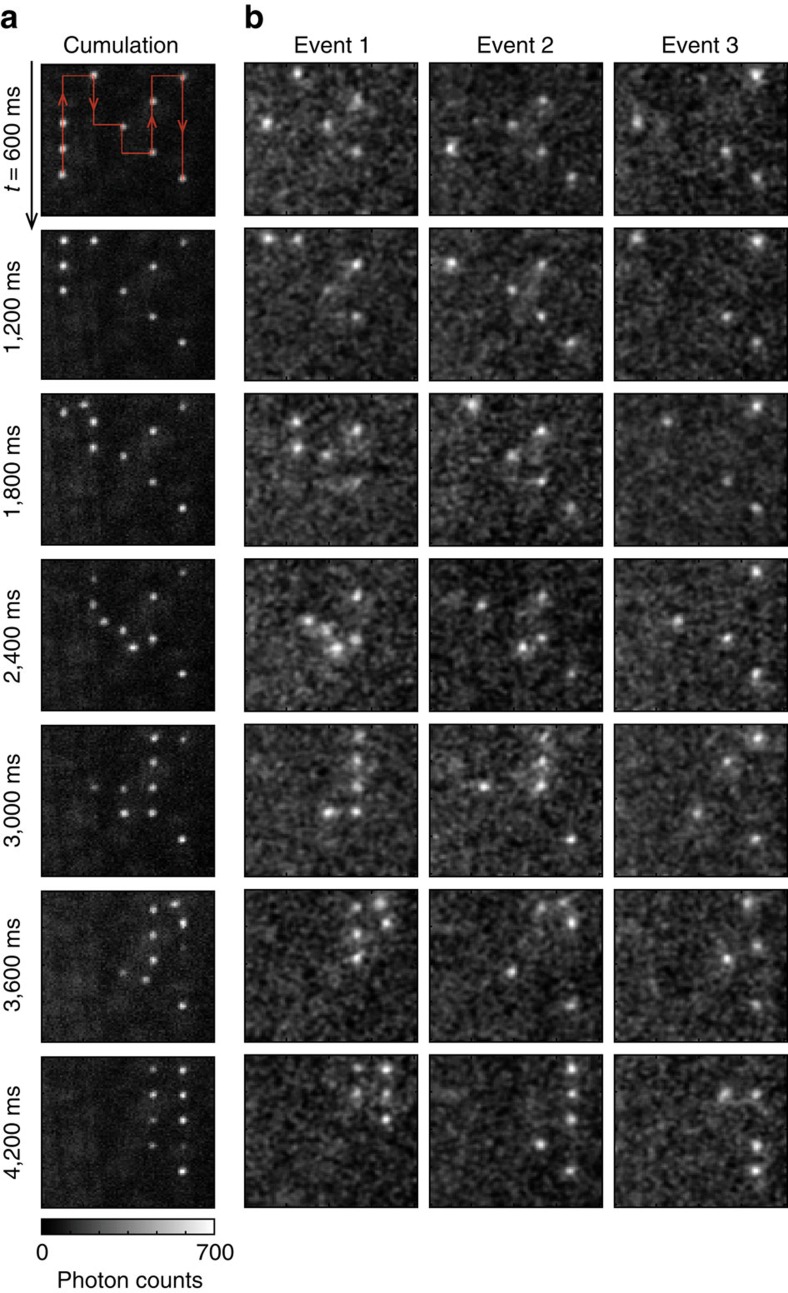
Single-atom transport example. (**a**) Transient cumulative images of 500 snapshots showing that nine atoms move the predefined path (red arrows) sequentially. (**b**) Three selected single events having four atoms or more. Every snapshot represents the same 26 × 26 μm^2^ area. The images are Gaussian-filtered for clarity.

An image accumulating 500 snapshots from different loading events is shown in Fig. 1c. The three-dimensional (3D) molasses continuous imaging^10^,^28^ captured a snapshot every 60 ms with 50 ms exposure time, where the single-event atom visibility exceeded 99.99%. If *N*_t_ increase, the number of SLM pixels per single optical tweezer would decrease, thus degrading the optical tweezer shape and intensity regularity. In Fig. 1d, the deviation of the normalized peak intensity *δ* (the *y* axis) is given proportional to the number of tweezers *N*_t_ (the *x* axis) and inversely proportional to the beam waist *W*, that is, *δ*∝(*N*_t_−1)/*W*. This behaviour can be understood as following: the peak intensity of each tweezer follows the binomial distribution *B*(*M*, 1/*N*_t_), where 1/*N*_t_ is the success probability and *M*≈*W*^2^ is the number of active SLM pixels, so the normalized variance is given by 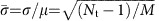, where the mean and variance are *μ*=*M*/*N*_t_ and *σ*^2^=*M*(*N*_t_−1)/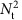, respectively. In addition, there are 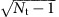 events that equally contribute to this normalized variance, so we get *δ*∝(*N*_t_−1)/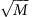
∝(*N*_t_−1)/*W*. To maintain an acceptable quality of the optical tweezers for the experiments, we chose *N*=9 and *W*=2 mm (the beam waist), limited by the laser power and relay optics dimensions, respectively. In our experiment, the standard deviation of the normalized peak intensity below 0.02 is tested, and a setup with a bigger laser power would support more optical tweezers. Note that, because of the 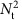 power-scaling, diffused light will eventually wash out the tweezer potential and the maximally available tweezers in our method is estimated to be 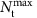
=100. Figure 1e shows an array-rearrangement demonstration. The series of images each accumulating also 500 snapshots of the individual experiments demonstrates that the initially prepared a 3-by-3 square array of single atoms with a spacing of *d*=4.5 μm expands to an array of twice the lattice spacing of 2*d*. Figure 2 shows a single-atom transport example; in this case, *N* single atoms are moved from an initial array along a predefined path. Because each atom moves in 2D with parameters (*X*_*i*_, *Y*_*i*_) for *i*=1 to *N*=9, the total degrees of freedom of movement is 2*N*=18.

### Single-atom array synthesis demonstration

**Figure 3:**
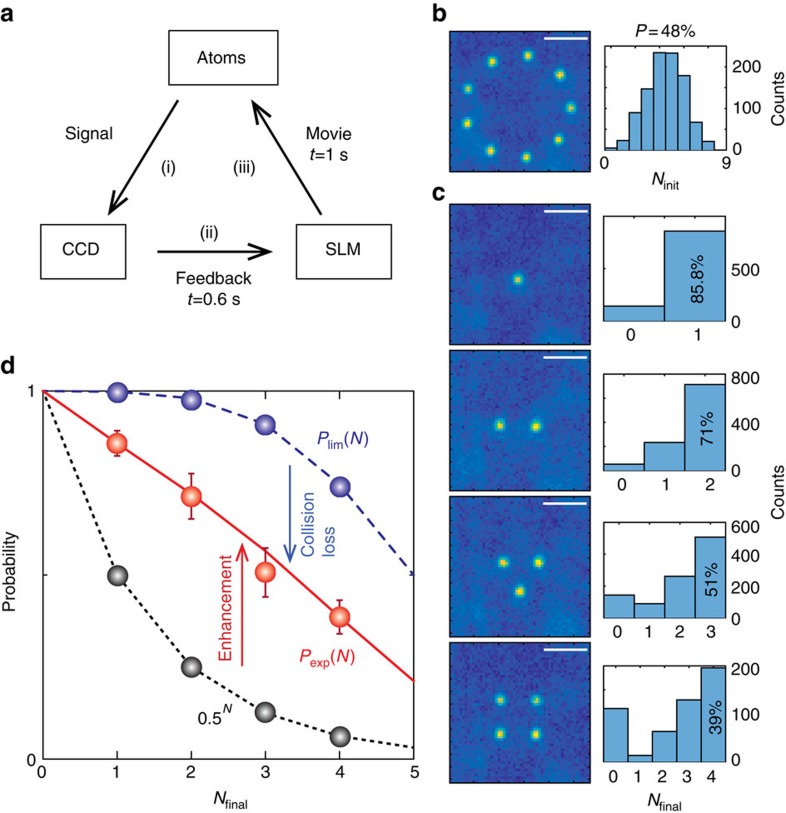
*In situ* single-atom array synthesis. (**a**) The feedback-control loop sequence: (i) signal gathering and processing with a CCD, (ii) state resolving and solution finding, (iii) solution execution with a SLM. (**b**) The cumulative image of initially loaded *N*=9 atoms (left), accompanied by the corresponding number histogram (right), where the scale bar size is 5 μm. (**c**) From the initial nine sites, one, two, three and four atoms are rearranged through single feedback loop. (**d**) Success probabilities of the atom rearrangement: *P*=0.5 (black dotted line), experiments (red line and data points) and theoretical limit (blue dashed line). The error bar depicts the standard deviation of time-binned 100 events.

A proof-of-principle demonstration of the *in situ* single-atom array synthesis is presented in Fig. 3. In a three-step feedback loop of initial atom loading in *N*_init_=9 sites, readout, and rearrangement, as shown in Fig. 3a, an atom array of *N*_final_=1, 2, 3 or 4, is produced. After the atoms are initially loaded at the sites, the first computer checks the occupancy of each site by reading out the electron multiplying charge-coupled device (CCD) images. A 9-bit binary information that represents the occupancy is then sent to the second computer, which has a look-up table of atom rearrangement trajectories, each stored in DRAM as a sequence of 30 holograms, between all possible initial and final pairs of atom arrays. It takes 0.6 s from the readout to the retrieval of an appropriate trajectory (note that, using a fast graphic-processing unit can greatly reduce this time and also make the look-up table unnecessary^29^). Then, the second computer sequentially loads the holograms to the SLM at a speed of 30 frame-per-second to move the atoms along the trajectory. The initial, and four types of final cumulative images are represented by the atom number histograms in Fig. 3b,c. The individual final images are independent experiments that form one, two, three or four atom arrays out of nine.

Compared with the binomial distribution of the initial histogram, the final histogram in Fig. 3d has non-Poissonian distribution. The loading efficiency curves of the final arrays are presented with red points for the data from the number histograms of at least 500 events. The black dotted line follows 0.5^*N*^, which is the loading efficiency in the collisional blockade regime, and the blue dashed line follows the cumulative binomial distribution given by


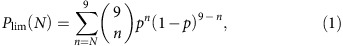


and the red line is


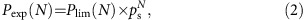


where *p*=0.48 is the initial loading probability and *p*_s_=0.86 is the experimental (moving) success probability from the fitting. 1−*p*_s_ is composed of the background collisional and moving losses. During the entire feedback process, the background collisional loss is estimated to be 0.13 and the moving loss is estimated to be 0.01. The moving success rate, as expected, has high fidelity and a deterministic transport has been reliably completed. The *N*_final_=4 case exhibits sixfold enhancement in loading efficiency, compared with the *P*=0.5 collisional blockade regime.

## Discussion

**Figure 4:**
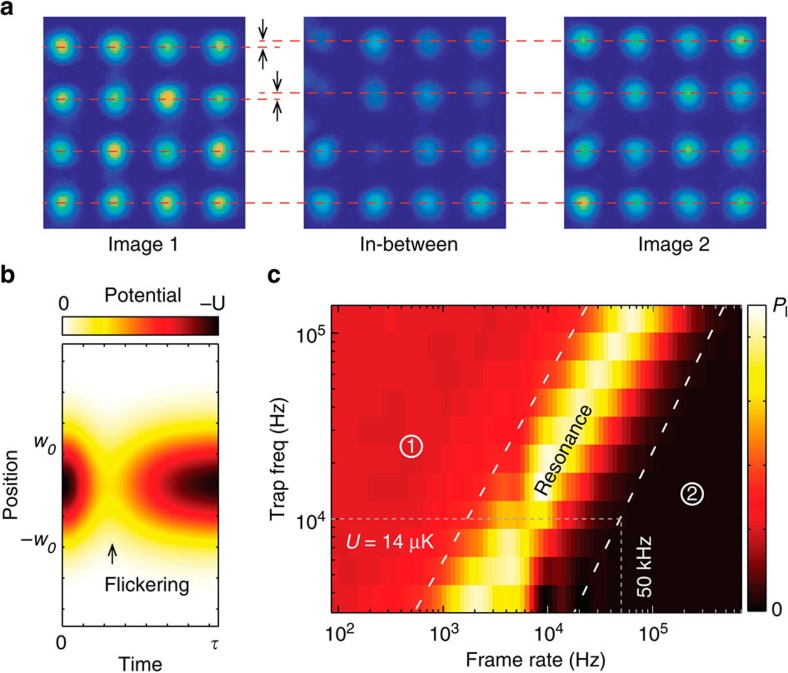
Intensity flicker of IFTA transport. (**a**) Stroboscopic measurement of optical images of the trap array. Two different images are generated using an IFTA and the LC-SLM. The two upper rows in the second and third frames (right) are slightly shifted up, by the gaps indicated by the arrows, from the first frame (left). (**b**) The transient potential for the trap loss simulation. The trap waist is *w*_o_=1.14 μm, the transient time 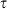
=1/*f* where *f* is frame rate, and the displacement *w*_o_/18. The colour scale is normalized by the peak potential, *U*. (**c**) Trap loss landscape by the flickering potential (**b**). The colour scale normalized by *P*_*l*_ represents the loss probability at time 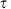, which varies by 0.005–0.04 according to the initial trap condition (*T*/*U*=1/18∼1/12).

The physics behind this demonstration is the capability of holographic optical tweezers to sustain trapped atoms while the hologram is being actively updated, but this has been considered impossible because of intensity flickering^30^,^31^. If conventional IFTAs are used to generate individual holograms to form the required optical potentials, the frame-to-frame evolution does not necessarily maintain an appropriate in-between potential (see Fig. 4a). While the intensity flickering is not an issue for macroscopic particles suspended in a solution^32^, microscopic particles (for example, atoms) do not wait until the missing potential recovers or cannot resist excessive displacement heating. Even with a fast device such as a digital micromirror device (50 kHz frame)^33^ or for ultracold atoms^34^, a large portion of the trapped atoms is lost. The trap loss simulation (see Methods) performed as a function of the frame rate, *f*, of the device and the trap frequency, 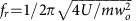, supports the idea that the intensity flickering hinders the trap stability (see Fig. 4b,c). In particular, a constant loss exists because of the significant intensity flickering in the adiabatic region (*f*_*r*_≫*f*, Region 1 in Fig. 4c). In the non-adiabatic region (*f*_*r*_<*f*, Region 2 in Fig. 4c), single steps do not lose atoms (because the intensity flickering is fast enough); however, in this region, either the atoms boil up fast via displacement heating or current technologies are not applicable. The holographic transport of single atoms, therefore, requires an alternative algorithmic approach (this work) to reduce the intensity flickering.

**Figure 5:**
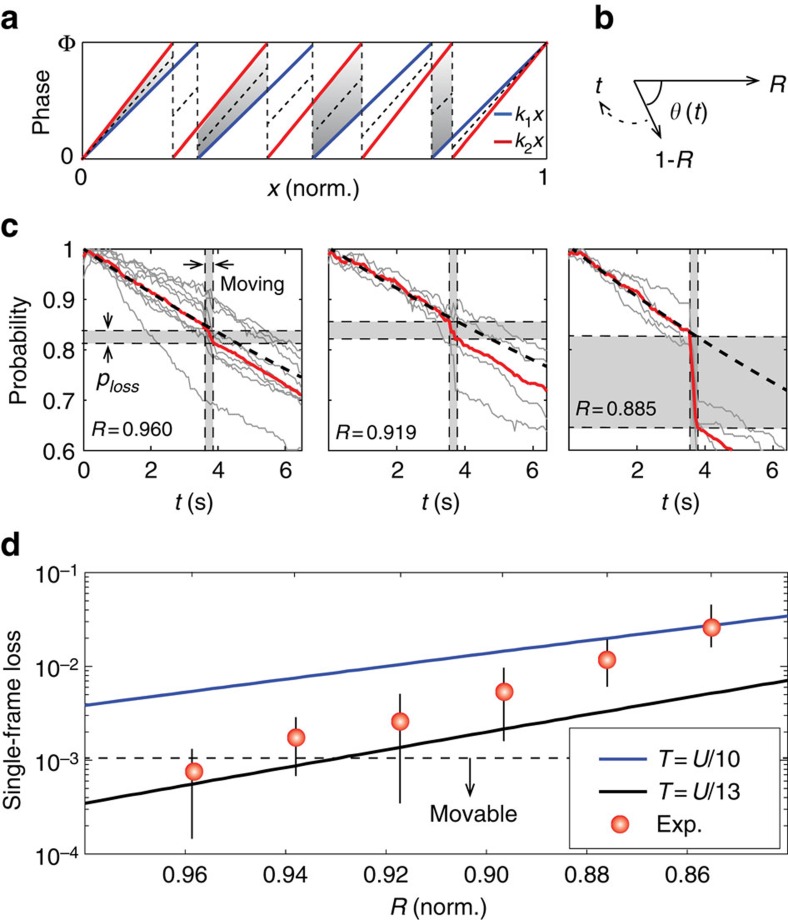
Transport loss mechanism. (**a**) Schematic representation of modulated two linear phases. The *x* axis is normalized to 1. The sum of the shaded regions is *R* which is proportional to field strength. (**b**) Transient vectorial representation of the flicker effect. (**c**) The experimentally measured loss probabilities are shown for three different tweezer displacements Δ*x*=180, 360 and 520 μm, respectively. Each data point (grey) is the average of 1,000 experimental runs, the red solid line is the average value of the data points, and the black dashed line is the loss without the displacement. The *x* axis is the time-lapse after the array loading, and the *y* axis is the single-atom survival probability measured using 1,000 cumulative events and normalized to 1. (**d**) Single-frame losses measured for various *R*s. The red circles are extracted single-frame data from **c** and the error bars represent the standard deviations of the different sites. The solid lines are from the adiabatic intensity flicker model (see Methods for more details).

The feature of the LC-SLM most strongly coupled to the intensity flicker is the finite modulation depth Φ (=2*π*). When a linear phase gradually changes from *k*_1_*x* to *k*_2_*x*, as depicted in Fig. 5a, certain regions (shaded) are flicker-free (because there are no phase jump), but the rest are not. An ideal flicker-free evolution could be achieved with infinitesimal change of Δ*k*, but it would then take infinite time to transport atoms. In experiment with a finite Δ*k*, we may quantify the intensity flicker by comparing the shaded region (denoted by *R* that is the field amplitude for a tweezer, normalized to one) with the unshaded region (denoted by 1−*R*). When the phase evolves from *k*_1_*x* to *k*_2_*x*, the fields from shaded region and unshaded region interfere destructively, because the unshaded region experiences Φ phase jump while the shaded region is stationary. In Fig. 5b, the arrows represent *R* (stationary) and 1−*R* (with a transient angle *θ*), respectively. Then, the peak intensity of an optical tweezer varies between 1 (the vector-sum maximum) and (2*R*−1)^2^ (the minimum), so the difference between them, that quantifies the intensity flicker, is given by 4*R*(1−*R*). In Fig. 5c, the survival probability change of the loaded atoms on the traps is shown. Between the two time-lapses for natural decays, the hologram movie was played to displace the optical tweezer, while snapshots were being taken in every 60 ms. Each measured atom decay curve shows the transport loss due to the intensity flicker induced by the 5 μm displacement of the optical tweezer. The sudden probability drop, *p*_loss_, is measured for each *R* value, and, from the measurement, the single-frame loss probability, *P*_l_=1−(1−*p*_loss_)^1/*n*^, where *n* is the number of frames in each displacement, is obtained as in Fig. 5d. Note that 1−*R* is proportional to the displacement step, Δ*x*. For example, *R*=0.96 and 0.94 correspond to Δ*x*=180, and 270 nm, respectively. When the result is compared with the adiabatic intensity flickering model (see Methods), the measured atom temperature agrees well with the temperatures of the two theory lines, 107 and 140 μK, respectively, in Fig. 5d. The agreement, therefore, supports that the dominant moving loss mechanism is the intensity flicker. Because the theory predicts that the single-frame loss can be exponentially decreased as a function of *R*, the high-fidelity holographic transport is achievable: for example, when we consider an empirically defined movable region of *R*>0.96, which is about 180 nm step, the fidelity after a 5 μm transport can exceed ∼0.99. Note that the deviation of data from the theory line at smaller *R* may come from stray B-field and the vector light shift of the optical tweezer, both of which cause force on atoms while the molasses is being turned on. As *R* is smaller, the trapped atoms are more vulnerable to the force and thus the loss has more increasing tendency.

Our method can be further improved by increasing the number of atoms and the efficiency of initial loading. Besides simply increasing the number of dynamic holographic tweezers, one may use a passive diffractive optical element (DOE) in addition. A commonly available 6-by-6 passive DOE, for example, can capture *N*=9 atoms with a probability exceeding 99.8% at *P*=0.48, so subsequently an active element (SLM) can pick up and move them by taking the full advantage of ‘all' available SLM tweezers. Also, the more stable traps, such as passive DOE or acousto-optic modulators, can be used to store them for an extended time^35^,^36^. (Note that the acousto-optic modulator-based method for 2D transportation uses *N*_1_+*N*_2_ diffracted beams to make an array of *N*_1_ × *N*_2_ tweezers, so the moving degrees of freedom in that case is limited by *N*_1_+*N*_2_.) Furthermore, lowering the background collision can further improve the performance: in a low-pressure (10^−11^ Torr) chamber that allows *p*_s_=0.97, the probability of creating a completely packed 3-by-3 atom array is expected to be as high as 80%.

In summary, the analytic approach to optical potential design has demonstrated the holographic transport of single-atom arrays. The systematic analysis of intensity flicker enabled the moving loss to be parameterized; thus, we could find and achieve the deterministic transport regime using a holographic method. An individual atom has its own degrees of freedom in the image plane; thus, total moving degrees of freedom of 2*N* was achieved, which demonstrates unprecedented space controllability. Furthermore, overlapping Fresnel lens pattern^37^ can transport the array in the axial direction, suggesting that total 3*N* degrees of freedom is also possible. We also formed an *in situ* feedback loop for atom array rearrangement, which is a proof-of-principle demonstration of high-fidelity atom array preparation. The possible application is not limited to the deterministic array preparation but may be extended to many-body physics with arranged atoms and coherent qubit transports.

## Methods

### Experiments

The active holographic device was a liquid-crystal SLM (HOLOEYE, PLUTO), a reflective phase modulator array of 1,920 × 1,080 pixels with an 8 μm^2^ pixel size and the first-order diffraction efficiency was ∼50%. The far-off-resonant trap (FORT) beam of *P*_total_=1.1 W from a Ti:sapphire continuous-wave laser (M Squared, SolsTiS) was tuned at *λ*=820 nm to illuminate the SLM with a beam radius of *W*=2 mm in a near-orthogonal incident angle. The diffracted beam from the SLM was imaged onto the intermediate image plane by an *F*_1_=200 mm lens, and then re-imaged onto the entrance pupil of the objective lens by a second lens (doublet, *F*_2_=200 mm). The objective lens (Mitutoyo, G Plan Apo) was an infinity-corrected system, having a focal length of *F*_3_=4 mm, a numerical aperture of NA=0.5, and a long working distance of 16 mm with 3.5 mm-thick glass-plate compensation. Then the given laser power was able to sustain up to 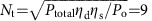 optical tweezers with a trap depth of *U*=1.4 mK, where the diffraction efficiency was *η*_d_=0.5, the optical system loss *η*_s_=0.5, and the optical power *P*_o_=3.4 mW.

The cold ^87^Rb atom chamber was a dilute vapour glass cell in a constant pressure of 3 × 10^−10^ Torr. It had four 100 × 40 mm^2^ clear windows with a thickness of 3.5 mm. The ^87^Rb atoms from a getter were captured by a six-arm 3D magneto-optical trap with a beam diameter of 7.5 mm (1/*e*^2^), a detuning of −18 MHz from the 5*S*_1/2_(*F*=2)→5*P*_3/2_(*F* ′=3) hyperfine transition, and d*B*/d*z*=15 G cm^−1^. After an initial magneto-optical trap loading operation for 2.8 s, the atom density became ∼10^10^ cm^−3^ (equivalent to 0.2 atoms per single trap volume), so the −46-MHz detuned 3D molasses and the FORT were overlapped for 200 ms to achieve the collisional blockade regime of the *P*=0.5 filling probability in every site^10^. After this, the magnetic-field gradient and the molasses were turned off for 100 ms to dissipate residual cold atoms and then the 3D molasses were turned back on for continuous imaging. The scattered photons were collected by the same objective lens *F*_3_ and imaged onto the CCD (Andor, iXon3 897) through the lens *F*_2_ with an overall efficiency of *η*_c_=0.02. The image plane of 26 × 26 μm^2^ was captured as a snapshot in every 60 ms. The trap lifetime was measured as 12 s, consistent with the effect of the background gas collision.

### Single-atom detection

The scattering cross-section is given by *σ*=*σ*_0_/[1+4(Δ/Γ)^2^+*I*/*I*_sat_], where the resonant cross-section is *σ*_0_=2.907 × 10^−9^ cm^2^, the natural line width Γ=5.746 MHz, the saturation intensity *I*_sat_=1.669 mW cm^−2^, the detuning Δ=−100 MHz (Stark shift is considered), and the intensity *I*=27 mW cm^−2^. Each atom in an optical tweezer emits 2.87 × 10^5^ photons per second. With the overall detection efficiency of 0.02 and the exposure time of 50 ms, we collect 280 photons per atom. It corresponds to a signal-to-noise ratio >5 for the CCD. When a Gaussian noise is assumed, the theoretical discrimination probability between zero and single atom is given with 5*σ* significance or 99.99995%. In the experiments, a background photon noise exists, but the histogram shows the success probability exceeding 99.99%.

### Heating in optical tweezers

There are several heating sources for trapped atoms including the FORT scattering, the intensity noise, the beam pointing fluctuation; however, none of them are a significant heating source. The heating caused by the photon scattering from FORT is estimated as 7 μK s^−1^ at the peak intensity, which is negligible in our trap. The intensity fluctuation (Δ*I*) and beam pointing fluctuation (Δ*w*_o_) by the LC-SLM voltage update are up to 6% and 5%, respectively; its noise spectrum, however, is less than 500 Hz. The parametric heating has harmonic resonances at *ω*_*r*_/2*n*^38^,^39^, which are far from 500 Hz. The quantitative heating rate has not been estimated; however, any atom loss difference is not observed during the one second transport without the molasses. Empirically the low-frequency noise does not degrade the trap capability in our 1.4 mK deep trap. Utilizing an intensity feedback control to the diffraction beam will diminish the intensity fluctuation^40^; thus, a lower trap depth would be achievable.

### Dynamic range and resolution of the control space

The optical tweezers are separated from the zeroth order diffraction by *X*_01_=*F*_1_*F*_3_/*F*_2_ × *k*_1_/*k*>5 μm to avoid the cross-talk, which sets the lower limit 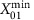
=5 μm of the dynamic range of the control space. The upper limit is empirically given by 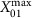
=45 μm, because the diffraction efficiency decreases as *k*_1_ increases. Note that the entrance pupil diameter of the objective lens is D∼2NA*F*_3_ and the initial beam diameter 2 *W*=4 mm at the SLM is (de)magnified by the ratio *F*_2_/*F*_1_=1 to fit with *D* for optimal performance, which results in *X*_01_=*D*/2N*A* × *k*_1_/*k*, independent of the focal lengths of the system. In our experiment, a safe working area of 26 × 26 μm^2^ is used for the optical tweezer patterns and the imaging plane. The discrete phase induces a beam steering error^41^ but the amount of the error is only 4 nm in our system with 256 grey levels. Thus the resolution is limited by 4 nm, which is much smaller than the long term drift (100 nm) and the LC-SLM refresh fluctuation (100 nm).

### Adiabatic intensity flicker model

The probability for an atom initially trapped in a potential *U* to escape from an adiabatically lowered potential *U*′ is approximately given by


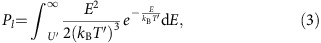


where 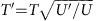 is the temperature of the Boltzman distribution^26^. In our experiment, the lowest trap potential is given by *U*′=(2*R*−1)^2^*U*. The solid lines in Fig. 5d are the numerically obtained results of equation (3) for *T*=*U*/13 and *T*=*U*/10, respectively.

### Intensity flicker estimation

The finite modulation depth (0≤*ϕ*≤Φ) of the SLM phase restricts the ideal linear phase to a modulated phase in a sawtooth shape. We consider two SLM phases *ϕ*_1_(*x*)=mod(*k*_1_*x*+Φ/2, Φ) and *ϕ*_2_(*x*)=mod(*k*_2_*x*+Φ/2, Φ), where Φ∈2*πN*, *x*∈[−*D*/2, *D*/2], *D*=4.6 mm is the size of the active SLM window, and we assume *k*_1_≤*k*_2_ without loss of generality. The phase evolution from *ϕ*_1_(*x*) to *ϕ*_2_(*x*) is then given by 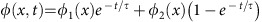 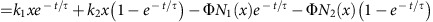, where *N*_1,2_(*x*)=[*k*_1,2_*x*/Φ] is a function defined with the Gauss' symbol [*x*]=*x*−mod(*x*) and 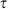 denotes the response time. The condition *N*_1_(*x*)=*N*_2_(*x*) defines the flicker-free evolution regions (the shaded regions in Fig. 5a). In our experiment, the flicker-free regions are divided into two regions respectively satisfying *N*_1_(*x*)=*N*_2_(*x*) and *N*_1_(*x*)=*N*_2_(*x*)−1, because *R* is large enough for an atom transport or (*k*_2_−*k*_1_)(*D*/2)<2Φ. As a result, *R* defined by the sum of the flicker-free regions divided by *D*/2 is given by


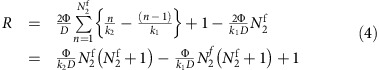


for the case of *N*_1_(*D*/2)=*N*_2_(*D*/2)≡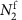 and


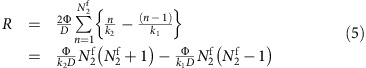


for the case of *N*_1_(*D*/2)=*N*_2_(*D*/2). The *R* in equation (4) can be further simplified to


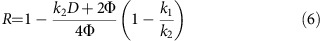


under the assumption *N*_1_=*N*_2_≈*k*_2_*D*/2/Φ. The obtained analytic result for the *R* is within 2% difference from the actual numerical value estimated by considering the circular active SLM window, the Gaussian beam profile of the optical tweezers, the 2D nature of the *k*1 and *k*2, and the discrete pixel size of the SLM. Within the experimental variation of *k*1 and *k*2, *R* varies from 0.86 to 0.96 as shown in Fig. 5d.

### Single-frame displacement limit

The maximum single-frame displacement Δ*X*_01_=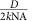
Δ*k* is also given as a function of *R*. When equation (6) is expressed with *k*_2_*D*=2*k*NA*X*_01_ and 1−*k*_1_/*k*_2_=Δ*X*_01_/*X*_01_, we obtain


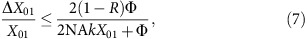


so the single-frame displacement is proportional to 1−*R*.

### The trap loss simulation in Fig. 4b,c

For a pair of initial and final trap potentials, which have *N* trap sites, two phase holograms are calculated using Gerschberg–Saxton algorithm, respectively. Some of the sites in the initial trap potential are separated by 1/18*w*_o_ from the final trap potential. The in-between holograms are constructed by *ϕ*_1_*e*^−*t*/*τ*^+*ϕ*_2_(1−*e*^−*t*/*τ*^), which generate the transient behaviour of a single trap potential as shown in Fig. 4b. Then, the trajectories (*p*, *q*) of the 1D classical Hamiltonian equation of motion calculated by the symplectic Euler method are used to estimate the trap loss probability in Fig. 4c, where we use a loss criteria of |*q*(*t*)|>2.5*w*_o_ and the initial energy and positions are sampled using the Monte-Carlo method.

### Data availability

The data that support the findings of this study are available from the corresponding author upon request.

## Additional information

**How to cite this article:** Kim, H. *et al*. *In situ* single-atom array synthesis using dynamic holographic optical tweezers. *Nat. Commun.* **7,** 13317 doi: 10.1038/ncomms13317 (2016).

**Publisher's note:** Springer Nature remains neutral with regard to jurisdictional claims in published maps and institutional affiliations.

## References

[b1] Schlosser N., Reymond G., Protsenko I. & Grangier P. Sub-poissonian loading of single atoms in a microscopic dipole trap. Nature 411, 1024–1027 (2001). 11429597 10.1038/35082512

[b2] Schlosser N., Reymond G. & Grangier P. Collisional blockade in microscopic optical dipole traps. Phys. Rev. Lett. 89, 023005 (2002). 12096994 10.1103/PhysRevLett.89.023005

[b3] Karski M. . Quantum walk in position space with single optically trapped atoms. Science 325, 174–177 (2009). 19589996 10.1126/science.1174436

[b4] Preiss P. M. . Strongly correlated quantum walks in optical lattices. Science 347, 1229–1233 (2015). 25766229 10.1126/science.1260364

[b5] Beugnon J. . Quantum interference between two single photons emitted by independently trapped atoms. Nature 440, 779–782 (2006). 16598253 10.1038/nature04628

[b6] Bakr W. S., Gillen J. I., Peng A., Folling S. & Greiner M. A quantum gas microscope for detecting single atoms in a Hubbard-regime optical lattice. Nature 462, 74–77 (2009). 19890326 10.1038/nature08482

[b7] Miroshnychenko Y. . An atom-sorting machine. Nature 442, 151–151 (2006). 16838011 10.1038/442151a

[b8] Isenhower L. . Demonstration of a neutral atom controlled-NOT quantum gate. Phys. Rev. Lett. 104, 010503 (2006). 10.1103/PhysRevLett.104.010503 20366355

[b9] Kaufman A. M. . Two-particle quantum interference in tunnel-coupled optical tweezers. Science 345, 306–309 (2014). 24968938 10.1126/science.1250057

[b10] Nelson K. D., Li X. & Weiss D. S. Imaging single atoms in a three-dimensional array. Nat. Phys. 3, 556–560 (2007).

[b11] Wang Y., Zhang X., Corcovilos T. A., Kumar A. & Weiss D. S. Coherent addressing of individual neutral atoms in a 3D optical lattice. Phys. Rev. Lett. 115, 043003 (2015). 26252680 10.1103/PhysRevLett.115.043003

[b12] Nogrette F. . Single-atom trapping in holographic 2D arrays of microtraps with arbitrary geometries. Phys. Rev. X 4, 021034 (2014).

[b13] Xia T. . Randomized benchmarking of single-qubit gates in a 2D array of neutral-atom qubits. Phys. Rev. Lett. 114, 100503 (2015). 25815916 10.1103/PhysRevLett.114.100503

[b14] Fung Y., Carpentier A., Sompet P. & Andersen M. Two-atom collisions and the loading of atoms in microtraps. Entropy 16, 582–606 (2014).

[b15] Lester B. J., Luick N., Kaufman A. M., Reynolds C. M. & Regal C. A. Rapid production of uniformly filled arrays of neutral atoms. Phys. Rev. Lett. 115, 073003 (2015). 26317718 10.1103/PhysRevLett.115.073003

[b16] Bakr W. S. . Orbital excitation blockade and algorithmic cooling in quantum gases. Nature 480, 500–503 (2011). 22193104 10.1038/nature10668

[b17] Sherson J. F. . Single-atom-resolved fluorescence imaging of an atomic Mott insulator. Nature 467, 68–72 (2010). 20720540 10.1038/nature09378

[b18] Weiss D. S. . Another way to approach zero entropy for a finite system of atoms. Phys. Rev. A 70, 040302 (R) (2004).

[b19] Vala J. . Perfect pattern formation of neutral atoms in an addressable optical lattice. Phys. Rev. A 71, 032324 (2005).

[b20] Lengwenus A., Kruse J., Schlosser M., Tichelmann S. & Birkl G. Coherent transport of atomic quantum states in a scalable shift register. Phys. Rev. Lett. 105, 170502 (2010). 21231030 10.1103/PhysRevLett.105.170502

[b21] Kuhr S. . Coherence properties and quantum state transportation in an optical conveyor belt. Phys. Rev. Lett. 91, 213002 (2003). 14683295 10.1103/PhysRevLett.91.213002

[b22] Kaufman A. M. . Entangling two transportable neutral atoms via local spin exchange. Nature 527, 208–211 (2015). 26524533 10.1038/nature16073

[b23] Xu P. . Interaction-induced decay of a heteronuclear two-atom system. Nat. Commun. 6, 7803 (2015). 26199051 10.1038/ncomms8803 PMC4525158

[b24] Dorner U., Calarco T., Zoller P., Browaeys A. & Grangier P. Quantum logic via optimal control in holographic dipole traps. J. Opt. B Quant. Semiclass. Opt. 7, S341–S346 (2005).

[b25] Sortais Y. R. P. . Diffraction-limited optics for single-atom manipulation. Phys. Rev. A 75, 013406 (2007).

[b26] Tuchendler C., Lance A. M., Browaeys A., Sortais Y. R. P. & Grangier P. Energy distribution and cooling of a single atom in an optical tweezer. Phys. Rev. A 78, 033425 (2008).

[b27] Montes-Usategui M., Pleguezuelos E., Andilla J. & Martin-Badosa E. Fast generation of holographic optical tweezers by random mask encoding of Fourier components. Opt. Express 14, 2101–2107 (2006). 19503542 10.1364/oe.14.002101

[b28] Miroshnychenko Y. . Continued imaging of the transport of a single neutral atom. Opt. Express 11, 3498–3502 (2003). 19471484 10.1364/oe.11.003498

[b29] Shimobaba T., Ito T., Masuda N., Ichihashi Y. & Takada N. Fast calculation of computer-generated-hologram on AMD HD5000 series GPU and OpenCL. Opt. Express 18, 9955–9960 (2010). 20588849 10.1364/OE.18.009955

[b30] McGloin D., Spalding G. C., Melville H., Sibbett W. & Dholakia K. Applications of spatial light modulators in atom optics. Opt. Express 11, 158–166 (2003). 19461719 10.1364/oe.11.000158

[b31] He X., Xu P., Wang J. & Zhan M. Rotating single atoms in a ring lattice generated by a spatial light modulator. Opt. Express 17, 21007–21014 (2009). 19997339 10.1364/OE.17.021007

[b32] Curtis J. E., Koss B. A. & Grier D. G. Dynamic holographic optical tweezers. Opt. Commun. 207, 169–175 (2002).

[b33] Muldoon C. . Control and manipulation of cold atoms in optical tweezers. New J. Phys. 14, 073051 (2012).

[b34] Boyer V. . Dynamic manipulation of Bose-Einstein condensates with a spatial light modulator. Phys. Rev. A 73, 031402 (2006).

[b35] Endres M. . Cold Matter Assembled Atom-by-Atom.. http://arxiv.org/abs/1607.03044 (2016).

[b36] Barredo D. . An atom-by-atom assembler of defect-free arbitrary 2d atomic arrays. http://arxiv.org/abs/1607.03042 (2016). 10.1126/science.aah3778 27811285

[b37] Lee W., Kim H. & Ahn J. Three-dimensional rearrangement of single atoms using actively controlled optical microtraps. Opt. Express 24, 9816–9825 (2016). 27137595 10.1364/OE.24.009816

[b38] Jauregui R. Nonperturbative and perturbative treatments of parametric heating in atom traps. Phys. Rev. A 64, 053408 (2001).

[b39] Gehm M. E., O'Hara K. M., Savard T. A. & Thomas J. E. Dynamics of noise-induced heating in atom traps. Phys. Rev. A 58, 3914–3921 (1998).

[b40] McGovern M., Grunzweig T., Hilliard A. J. & Andersen M. F. Single beam atom sorting machine. Laser Phys. Lett. 9, 78–84 (2012).

[b41] Engstrom D., Bengtsson J., Eriksson E. & Goksor M. Improved beam steering accuracy of a single beam with a 1D phase-only spatial light modulator. Opt. Express 16, 18275–18287 (2008). 18958104 10.1364/oe.16.018275

